# A feminist ethos for caring knowledge production in transdisciplinary sustainability science

**DOI:** 10.1007/s11625-021-01064-0

**Published:** 2021-12-11

**Authors:** Rachel K. Staffa, Maraja Riechers, Berta Martín-López

**Affiliations:** Social-Ecological Systems Institute, Faculty of Sustainability, Leuphana University Lüneburg, Universitätsallee 1, 21335 Lüneburg, Germany

**Keywords:** Care, Transdisciplinary sustainability science, Feminist research, Transformative research, Reflexivity, Empowerment

## Abstract

Transdisciplinary Sustainability Science has emerged as a viable answer to current sustainability crises with the aim to strengthen collaborative knowledge production. To expand its transformative potential, we argue that Transdisciplinary Sustainability Science needs to thoroughly engage with questions of unequal power relations and hierarchical scientific constructs. Drawing on the work of the feminist philosopher María Puig de la Bellacasa, we examine a feminist ethos of care which might provide useful guidance for sustainability researchers who are interested in generating critical-emancipatory knowledge. A feminist ethos of care is constituted by three interrelated modes of knowledge production: (1) thinking-with, (2) dissenting-within and (3) thinking-for. These modes of thinking and knowing enrich knowledge co-production in Transdisciplinary Sustainability Science by (i) embracing relational ontologies, (ii) relating to the ‘other than human’, (iii) cultivating caring academic cultures, (iv) taking care of non-academic research partners, (v) engaging with conflict and difference, (vi) interrogating positionalities and power relations through reflexivity, (vii) building upon marginalised knowledges via feminist standpoints and (viii) countering epistemic violence within and beyond academia. With our paper, we aim to make a specific feminist contribution to the field of Transdisciplinary Sustainability Science and emphasise its potentials to advance this field.

## Introduction

Transdisciplinary Sustainability Science has increasingly been pursued over the last two decades to understand and transform the multiple sustainability crises societies are facing (Heinberg and Lerch 2010; IPBES 2019; IPCC 2014). With its explicit commitment to sustainability and intergenerational justice, Transdisciplinary Sustainability Science aims at fostering socially and environmentally sound human-nature interactions. A main modus operandi hereby is the integration of bodies of knowledge from various scientific and non-academic backgrounds (König 2017; Lang et al. 2012; Tengö et al. 2017). In doing so, Transdisciplinary Sustainability Science has been claimed to be a transformative science, that is a mode of science that not only analyses, but actively supports and accelerates sustainability transformations (Schneidewind et al. 2016; Vilsmaier and Lang 2014; Wiek and Lang 2016). However, truly transformative science requires a strong commitment to challenge unequal power structures and relations to design and navigate pathways towards more sustainable futures. Given that questions of power are inherent to processes of Transdisciplinary Sustainability Science (Fritz and Binder 2020; Fritz and Meinherz 2020; Hofmeister 2017), we suggest that Transdisciplinary Sustainability Science can extend its transformative potential through the commitment to a power-critical *feminist ethos of care* (see Box 1).

Feminist scholars have long pointed to the significance of care and have discussed relational ethics (e.g. Gilligan 1982; Held 2006; Kittay 1999; Noddings 1984), principles (e.g. Tronto 1993, 2013) and practices of care in their work (e.g. Maeve 1997; Mortlock 1996; Sherwood 1993). In particular, care is highly relevant in processes of knowledge production (Edwards and Mauthner 2012; Kingston 2019; Rose 1983; White and Bailey 2004). Also in recent academic discussions within Transdisciplinary Sustainability Science, broader attention has been given to a possible ‘relational turn’ of Sustainability Science (e.g. Mancilla García et al. 2020; Walsh et al. 2021; West et al. 2020), which points to the necessity of understanding human-nature relations as intrinsically power-laden, processual, and in need of care and stewardship by various actors (Bieling et al. 2020; Raatikainen et al. 2020; West et al. 2020), particularly by us researchers (Sellberg et al. 2021).

With our paper, we aim to contribute to the convergence of these two rich discourses (i.e. feminist research approaches and Transdisciplinary Sustainability Science) and emphasise the urgency of integrating power-critical feminist research approaches into the processual design of Transdisciplinary Sustainability Science (Hummel and Stieß 2017). Although other research fields, such as Environmental Justice and Political Ecology, have contributed tremendously to the study of unequal power relations in socio-ecological transformations using a feminist lens (e.g. Elmhirst 2015; Gaard 2017b; Rocheleau et al. 1996; Stein 2004), they did not explicitly discuss how to integrate power-critical feminist approaches into the processual design of Transdisciplinary Sustainability Science. In this paper, we concentrate on applying a specific framework, which is inspired by María Puig de la Bellacasa (2017)—a feminist philosopher and Science and Technology Studies (STS) scholar—to the field of Transdisciplinary Sustainability Science.

Drawing on her seminal work on a caring research ethos for transdisciplinary STS, we examine three modes of knowledge co-production for Transdisciplinary Sustainability Science, which seek to criticise and actively transform unequal power relations. *Thinking-with* encourages us to think beyond dualisms, to consider the multiplicity of society-nature relations, to open ourselves up to non-anthropocentric methodologies and to value affectivity in research. It also appeals to us to build a caring culture within research teams as well as trustful, empowering, diverse and long-term partnerships with non-academic actors. *Dissenting-within* points to the necessity of committed engagement with difference and conflict as well as critical reflection on positionality, normativity and hierarchies in power-laden research contexts. *Thinking-for* calls on us to include marginalised voices into research projects, but similarly to reflect on the dangers of appropriating these voices. Lastly, this mode of thinking commits us to acknowledge our privileges within academia and to work for the diversification of the academic landscape.

Box 1 Key terms and definitions **Appropriating**

Caring research requires us to reflect critically on the issue of representation and the inherent danger of appropriating the voices of our non-academic partners, i.e. to project our categories and worldviews onto their experiences, thereby, most often unwillingly, dismissing their unique knowledges and epistemologies (Dotson 2011). This act of silencing denies our partners the possibility of self-representation and complicates formulating empowering solutions with them (Spivak 1988).

**Care**

Etymologically, the word ‘care’, from the Old English ‘cearu’ translate into two meanings: an active one of attentiveness, regard, consideration and a passive meaning of worry, grief and anxiety (Moriggi et al. 2020a, b; Ahmed 2017). In this paper, we focus on the first meaning and define care as an attentive and power-critical commitment for the wellbeing of our world as composed of continually unfolding relationships and interdependencies between humans and all that is living and non-living (adapted from Puig de la Bellacasa 2017). Care is a situated and vital everyday maintenance practice, an affective engagement, as well as an ethico-political obligation to challenge unequal power relations.

**Ethos of care**

Ethos: the distinguishing practices and guiding beliefs of a person, group, or organisation (Merriam Webster Dictionary 2020). Ethos of care: An approach to morality that calls for putting at the centre the importance of context, interdependence, relationships and responsibilities (Held 2006; Koggel and Orme 2010). In this paper, we unpack the ethos of care approach for Transdisciplinary Sustainability Science by building on the three modes of knowledge co-production of thinking-with, dissenting-within and thinking-for (Puig de la Bellacasa 2017).

**Intersectionality**

The concept of intersectionality was first coined by black feminist scholar and civil rights activist Kimberlé Crenshaw (1989, 2017) to highlight the complex interplay of multiple systems of oppression (such as racism, sexism and classism) that affect the identities and experiences of marginalised individuals and groups differently. For a critical overview on depoliticizing and discriminatory tendencies in the intersectionality discourse, see Davis (2020) and Salskov (2020).

**Normativity**

Research done conforming to or based on norms (Merriam Webster Dictionary 2020). Sustainability Science explicitly acknowledges the aim for sustainability as normative context (while definitions and clarifications are, of course, debated) (Ziegler and Ott 2011).

**Positionality**

The role of the (multiple) self of the researcher, in terms of race, nationality, age, gender, disability, sexuality, social and economic status. This positionality may influence how and what data is collected, analysed and highlighted (Madge 1993; Rose 1997). Positionality can indicate the kind of power that enabled a certain kind of knowledge, instead of a universal scientific knowledge (Haraway 1988).

**Situated knowledges**

A concept highlighting that knowledge is always partial, contextual, and produced from a specific and fluid social position of the researcher who is required to critically interrogate their embeddedness in power-laden research processes, i.e. to make use of the practice of reflexivity (Haraway 1988).

The paper is structured as follows: First, we point to the critical-emancipatory potentials that a feminist perspective can offer as guidance for Transdisciplinary Sustainability Science. Second, we present the theoretical framework of care as developed by Puig de la Bellacasa (2017) that underpins a transformative research ethos. Third, we elaborate on how three modes of knowledge co-production—*thinking-with*, *dissenting-within* and *thinking-for*—can enrich processes of knowledge building in Transdisciplinary Sustainability Science. Fourth, we provide an outlook for practicing caring knowledge production in Transdisciplinary Sustainability Science research.

## Promising potentials for a feminist-infused transdisciplinary sustainability science

Over the past few years, feminist sustainability scientists have emphasised large overlaps of Transdisciplinary Sustainability Science and feminist research approaches. By rejecting the traditional notion of value-free, ahistorical, objective, and universal scientific knowledge, and instead of pointing to ‘situated knowledges’ (see Box 1) of different knowers (Haraway 1988), feminist research approaches and Transdisciplinary Sustainability Science have strongly critiqued the self-image of traditional science as a hierarchically superior knowledge system (Gottschlich and Katz 2016; Vilsmaier and Lang 2014). These fields of research seek to support the participation of civil society actors in research processes and aim at developing equitable relationships among them and between them and researchers (Hofmeister et al. 2013). The overall political and normative commitment of both fields is to transform present conditions and tackle societal problems through pursuing an integrative problem analysis and collaboratively discussing, negotiating, planning and implementing more sustainable pathways into the future (Gottschlich and Katz 2016).

Despite the convergences between the two research fields, it is feminist research that has long and strongly advocated for an inclusion of crucial perspectives on power, questions of domination and hierarchies within research processes. In our work, we have experienced, like others in the field (e.g. Fritz and Binder 2020; Ghosh 2020; Hofmeister 2017) that these inherent power dynamics remain often neglected and undiscussed when working through a research agenda. This is why we argue that Transdisciplinary Sustainability Science can extend its potential for sustainability transformations if it explicitly focusses on the identification, analysis and transformation of systems of oppression and unequal power structures, which produce marginalisation, exclusion, devaluation and discrimination against societal groups and natures (Gaard 2017a, b; Gottschlich and Katz 2016).

By integrating a power-critical feminist perspective, Transdisciplinary Sustainability Science could first, unveil and overcome dualist conceptualisations, which feminists have examined as a crisis-causing principle of thought in modern science (e.g. Plumwood 1993; Mathews 2017). In a not insignificant number of research projects, ‘nature’ is often still defined as humans’ other and remains reduced to resource and capital, whilst obfuscating the diverse interrelatedness of humans with natures (Gottschlich and Katz 2016). This dualistic conception of human/‘other than human’ has been considered as no longer tenable by feminist posthumanisms (Gaard 2017c), new materialisms (Wingrove 2015), and ecofeminisms (van den Berg 2018) and is, fortunately, being increasingly challenged within Sustainability Science (West et al. 2020).

Apart from the artificial conception of human/‘other than human’, feminist researchers have vehemently criticised the dualism of reason and emotion (Jaggar 1989; Little 1995) which remains an accepted paradigm within Transdisciplinary Sustainability Science. We as sustainability researchers routinely investigate deeply troubling, unjust and unsustainable developments and relations. Hence, it is very likely that we experience strong feelings of anger, hopelessness, despair, grief, or frustration, which are common among people who know too much about sustainability crises (Fischer and Riechers 2021; Haraway 2016). By often side-lining the expression of emotions to the private sphere, however, we defer to modern science’s spurious claim to objectivity and in doing so, we deny emotions their ability to act as driving forces for doing research. Considering emotions in the research process can create a deep awareness of the interdependence of, and empathy for, all beings in this world and help build a more stable foundation for effective action (Gottschlich 2017; Macy and Johnstone 2012).

Third, a joint consideration of affectivity in a research team can facilitate a caring academic culture that also allows for committed collaboration with non-academic actors (Iniesta-Arandia et al. 2016; Mountz et al. 2015). In fact, building strong affective research relationships can also assist in dealing with conflicts, which are inherent to transdisciplinary processes because sustainability problems involve diverse actors with competing interests, different interpretations of sustainability and multiple knowledge systems (Balvanera et al. 2017; König 2017). This is particularly important in light of institutional demands to produce smooth, but unrealistic win–win-solutions that might support our wish to avoid extensive discussion of conflicts of interest and underlying power relations (Hofmeister 2017).

Fourth, feminist scholars have fostered reflexivity as a key practice in research (e.g. Hesse-Biber and Piatelli 2012; Lumsden 2019); a practice less developed in Transdisciplinary Sustainability Science (but see e.g. Balvanera et al. 2017; Caniglia et al. 2021; Norström et al. 2020; Rosendahl et al. 2015). This power-critical practice involves disclosing the normative assumptions, values and concepts underlying the research processes, problematising differences in the status and effectiveness of different forms of knowledge at various research stages, as well as power differentials between non-academic actors and us scientists. Furthermore, it helps us to examine our positionality (see Box 1) and embeddedness in power-laden research contexts, interrogating the limits, pitfalls, or blind spots of our research (Lumsden 2019). By appreciating the normative, value-laden character of research processes and committing to foster social justice, we are more likely to give long-term, encompassing, and empowering decision-making competencies to marginalised non-academic actors—which still only a small number of Transdisciplinary Sustainability Science projects pursues (Brandt et al. 2013)—and actively bring neglected voices to the research and decision-making tables (Turnhout et al. 2012).

## A feminist ethos of care: Puig de la Bellacasa’s framework

To support Transdisciplinary Sustainability Science in producing more critical-emancipatory knowledge, we suggest a commitment of us sustainability scientists to a feminist *ethos of care*. Care has been a longstanding focus of feminist thinking and found application in diverse research fields, such as ethics and philosophy (Held 2009; Sander-Staudt 2019), critical psychology (Govrin 2014), political theory (McMillan 2017), justice (Held 1995), citizenship (Sevenhuijsen 1998), migration studies (Datta et al. 2010), economics and business (Ballet et al. 2018; Hamington and Sander-Staudt 2011), disability studies and activism (Kittay 2011), ethics for animal rights (Donovan and Adams 2007), food politics (Jarosz 2014), as well as sociologies and anthropologies of health work and sciences (Sturm 2004). It conceptually draws on a key theme in feminist ethics that aims to overcome atomistic ontologies and suggests “interdependency as the ontological state in which humans and countless other beings unavoidably live” (Puig de la Bellacasa 2017: 4). Acknowledging this reality requires us to understand processes of knowledge co-production as an intrinsically relational practice that necessitates care. As the way in which we pursue our research has “world-making effects” (Puig de la Bellacasa 2017: 30) and might be materialised in concrete policies, practices and institutions that shape processes towards more (un)sustainable futures, we are required to engage in practices of thinking with care to develop responses to sustainability crises (Sellberg et al. 2021).

Examining an ethos of care for Transdisciplinary Sustainability Science is inspired by feminist philosopher María Puig de la Bellacasa’s work on a caring research ethos for science and technology studies. Following Puig de la Bellacasa, we define care as an attentive and power-critical commitment for the wellbeing of our world as composed of continually unfolding relationships and interdependencies between humans and all that is living and non-living (Puig de la Bellacasa 2012). Care refers to a situated and vital *everyday maintenance practice* (often referred to as ‘care work’), an *affective engagement*, as well as an *ethico-political obligation* to challenge unequal power relations. We thus see care as “an essential feature of transformative thinking, politics and alternative forms of organizing” (Puig de la Bellacasa 2017: 8). Thereby, in research practices especially, care equals a political project and does not only fuel hope and imagination for transformative action with its commitment “to think about how things could be different if they generated care” (Puig de la Bellacasa 2017: 60); but also does care call for “ongoing efforts within existing conditions but without accepting them as given” (Puig de la Bellacasa 2017: 43). Care entails “not shying away from what is important to us” (Puig de la Bellacasa 2017: 11), even if power struggles might ensue (Haraway 2016).

Engaging with the classical feminist commitment to attend to power relations, inequalities and exclusions, Puig de la Bellacasa (2017) outlines three interrelated modes of transformative practices of knowledge co-production in the transdisciplinary research programme of science and technology studies: (1) thinking-with, (2) dissenting-within and (3) thinking-for. We apply these three modes, which together constitute a feminist research ethos of care, to the field of Transdisciplinary Sustainability Science. This is because we argue that these modes can expand the transformative potential of Transdisciplinary Sustainability Science by empowering us to use our daily work as a site for sustainability change (Leach 2013). In the next section, we elaborate on the application of these three modes. For an application-oriented summary of a caring feminist ethos in Transdisciplinary Sustainability Science, see Table 1.

**Table 1:** Non-comprehensive list for transdisciplinary sustainability scientists with a commitment to a feminist ethos for caring knowledge production: *thinking-with*, *dissenting-within* and *thinking-for*

Modes of knowledge production	Concrete actions to produce caring knowledge in transdisciplinary sustainability science
Thinking-with	Use relational concepts and frameworks to capture the interrelatedness of life. Similarly, account for the complexity and heterogeneity of your object(s) of study. Open yourself up to include ‘other than human’ methodologies into your research project. Allow strong emotions and feelings that may result from your daily work. Collectively build structures of mutual support within a research team, including collaborative writing and discussion of ideas, emotional support in times of struggle, different definitions of success, peer mentoring schemes, etc. Ensure that actors with a range of skills and multiple types of knowledge and expertise can participate in the project. Build trustful and respectful relationships with your project partners. Be aware that developing these relationships takes time! Nurture diverse communication and interpersonal skills to enable team building processes and high levels of interactivity. Refrain from scientific communication habits and translate your concepts and theories into everyday language. Give decision-making authority to non-academic partners and regularly discuss and reflect on the topic of co-determination and shared responsibility. Provide your partners with useful products that foster their empowerment.
Dissenting-within	Accept that there are no win–win-solutions, but always conflicting interests and values in transdisciplinary projects. Facilitate and participate in controversial discussions with non-academic partners and openly discuss divergent needs, interests, and expectations. Consider ‘asymmetrical reciprocity’ and listen ‘care-fully’ to your partners. Jointly define rules of engagement at the beginning of the project to create a productive culture of dispute. Utilise bridging concepts or boundary objects to reveal differences that might be transformed into a pluralistic understanding of the problem and for a spectrum of solution options. Ask for the implementation of self-managed conflict management programmes and for external support from consultants/mediators who might help us disclose and moderate conflicting objectives and interests. Critically reflect on your positionality and the power relations that suffuse the project. Acknowledge the differences and hierarchies in the status and effectiveness of different forms of knowledge, as well as status differences among the knowledge producers. Make your affiliations transparent and commit to particular struggles, because you cannot take care of everything.
Thinking-for	Show a commitment to analyse, critique and transform oppressive systems and produce knowledge for marginalised groups. Move knowledge from subjugated partners to the centre of your inquiry, given their disproportionate vulnerability and insider–outsider position. Critically reflect on whether you act as a spokesperson for these marginalised groups. Pursue a cautious and reflexive approach to speak *to* and *with* marginalised praxis partners. Stay with them and create spaces of trustful encounter. Compensate marginalised actors financially. Use methods through which they can speak in their own voice and express critique (e.g. photovoice, individual interviews, and discussion facilitators). Understand how systems of domination shape or limit research questions, methodological decisions, conceptual frameworks, models, assumptions and interpretations of data. Consider diversity within marginalised groups, use intersectional approaches to discuss unequal power relations and develop interventions that are responsive to the individual members of heterogeneous communities. Support marginalised communities in achieving their own critical standpoint by connecting them with other communities in struggle. Think about your relative privilege, become aware of exclusions within academia and actively work for the inclusion of underrepresented scholars.

## Thinking-with

### Embracing relational ontologies

*Thinking-with* particularly reminds us that knowledge production is an intrinsically collective enterprise of many participants that are mutually dependent on and interconnected with each other. To consistently *think-with* means becoming aware of this interrelatedness, so as to construct a different reality (Gottschlich and Katz 2018). Defining society-nature relations as the research framework of Transdisciplinary Sustainability Science (Becker et al. 2011; Hummel and Stieß 2017) follows Puig de la Bellacasa’s call “to confront and put into question the boundaries and cuts given in existing worlds” (2017: 72). Therefore, *thinking-with* demands from us to transcend hegemonic dualisms, that is, society/nature or culture/nature (e.g. West et al. 2020). It encourages us to acknowledge and transform the intricate relationships between societi*es* and natur*es*, which are not separate entities merely interacting with each other, but fluid, ever-changing assemblages, i.e. the assemblies or gatherings of various non-human and human agencies, which are symbolically, emotionally, and materially regulated in diverse ways (Tsing 2015).

Given the dynamic and processual character of sustainability transformations, society-nature relations are in a constant flux, situated in specific contexts, and continuously negotiated and challenged within power relations. This also means that multiple ways of understanding and transforming sustainable society-nature relations are possible (Riechers et al. 2021). As *thinking-with* is an acknowledgement of multiplicity which makes a “longing […] for fixed realities” impossible (Puig de la Bellacasa 2017: 72), this mode of thinking might call on us to nurture key skills, such as openness and “curiosity about the connected heterogeneities composing an entity, a body, a world, that troubles boundaries” (Puig de la Bellacasa 2017: 71). In Transdisciplinary Sustainability Science, it is widely agreed upon that accepting and productively engaging with the complexity that characterises the multiple ways societies relate to nature can reveal innovative and transformative solutions (Balvanera et al. 2017; Díaz et al. 2018; Fam et al. 2017; Thiem and Katz 2015).

### Relating to the ‘other than human’

*Thinking-with* also encourages us to include non-anthropocentric ontologies and therewith “enlarge our ontological and political sense of kinship and alliance” (Puig de la Bellacasa 2017: 73). Overcoming anthropocentric notions of sustainability requires us to challenge hierarchical constructions and acknowledge that ‘other than humans’ have meaning, power and agency of their own (Adelman 2018; Kaijser and Kronsell 2016; Moriggi et al. 2020a, b). Difficult debates on the intrinsic agencies, rights and values being conceded to ‘other than humans’ and what this means for concrete transformational strategies for research practice become inevitable. These debates, vibrantly held in the fields of feminist new materialisms (Truman 2020), (Indigenous) posthumanist thinking (Niccolini and Ringrose 2020; TallBear 2011), ecofeminism (Gaard 2017a), multispecies research (Swanson 2020) and environmental ethics and philosophy (Warren 2014), among others, need to be accorded much greater importance in Transdisciplinary Sustainability Science to foster both intra- and intergenerational and interspecies justice (Gaard 2015).

Overall, when relating to the ‘other than human’, a critical-emancipatory perspective not only analyses but also condemns unjust power relations and suffering. Developing the ability for compassion is the basis for seeing, feeling and helping to overcome the suffering of all beings, as well as for challenging power relations and constructed otherness by connecting through curiosity and emotion with ‘the other’ (Böhm and Ullrich 2019; Manemann 2014; Raatikainen et al. 2020). A feminist ethos of care thus calls for the reintegration of “affectionate knowing” (Puig de la Bellacasa 2017: 62) into scientific work which might also produce fundamental changes in how we relate to others and ourselves in the research process (Jax et al. 2018). Empirical studies of place-based experiential learning show that those learning experiments with transformative potential towards sustainability are those that integrate emotions and challenge power relations (Moriggi et al. 2020a, b). For example, Harmin et al. (2017) show that pedagogical experiences that allow individuals to engage deeply with non-human beings result in a change of paradigm, where nature is recognized as a sentient entity.

## Cultivating caring academic cultures

This reintegration of affectionate knowing into scientific work can also strongly affect academic cultures. Given the still dominant principles of objectivity and detachment that guide scientific practice (Corner et al. 2018), a feminist ethos of care denounces that the emotionality of researching difficult and sensitive topics remains a private issue for most researchers (Smith 1987; Gordon et al. 2019). There is an increasing, but still marginal number of sustainability scientists who openly ‘admit’ that their research affects them on an emotional level, thereby opposing the notion of the objective, value-free and detached truth-finder (Is This How You Feel 2020). Nevertheless, feelings, such as ‘ecological grief’ or ‘eco-anxiety’ (Cunsolo et al. 2018; Cunsolo et al. 2020; Plieninger et al. 2021), are often unavoidable side-effects of thinking with care (Puig de la Bellacasa 2017: 93) and may have powerful transformative capacity for system change (Ives et al. 2020; Riechers et al. 2019).

To enable such transformative capacity, developing and maintaining nurturing researcher communities characterised by mutual emotional support, collaboration, shared learning and accountability to team members, is vital (Iniesta-Arandia et al. 2016). These nurturing communities are a pre-condition of a knowledge production that can counter the increasing marginalisation and individualisation of caring arrangements within the neoliberal academic arena (Lipton and Mackinlay 2017; Mountz et al. 2015). The colonisation of knowledge production through neoliberal paradigms of efficiency, quantification and performance, has resulted in heightened competition, workload intensification and an audit culture, all of which place huge pressure on sustainability researchers (Fischer et al. 2012). However, as with eco-anxiety, these “hidden injuries of academia” (Bayfield et al. 2019: 7) are often borne silently and alone, with detrimental impacts on well-being and mental health (Powell 2017), as well as on our ability to address sustainability challenges (Paasche and Österblom 2019).

Individual self-care strategies, such as privately exercised mindfulness meditations or participation in time management workshops, might indeed reduce stress and improve mental health. However, they run the risk of solely placing responsibility on individual researchers to enact self-care and obfuscating structural barriers in academia (Care et al. 2021; Fischer et al. 2012). Structural barriers of academic institutions go beyond the reward and evaluation systems of researchers that most often foster individual and high-pace productivity, and include the lack of measures to establish a healthy work-life balance and a caring system for researchers (Sellberg et al. 2021). While there are some discussions on motherhood and how to foster an inclusive working environment in universities and research centres (Leventon et al. 2019), less is discussed when researchers (often women) have to take care of their elderly care-givers or family members with chronic diseases or disabilities. Even less is discussed when the researcher is the one disabled (but see Hartman 2019; Tuosto et al. 2020; and see Box 2 for a discussion on how a feminist ethos of care can contribute to support scholars with disabilities in Transdisciplinary Sustainability Science).

Box 2 Considering disability in Transdisciplinary Sustainability Science through a feminist ethos of care Berta Martín-López (Leuphana University of Lüneburg, Germany) and Aleksandra Kosanic (Liverpool John Moores University, United Kingdom).

Although all academic fields should aim to be at the forefront of progressive transformational change, in the case of Transdisciplinary Sustainability Science, the lack of consideration of disabilities in research projects, programmes and academic institutions is more critical than in other fields since it threatens its main goal of promoting sustainability and equity. “There are too many obstacles in academia for people who have physical or mental-health conditions—and who have much to offer to science” (Schaal 2018). As it happens with any discrimination, scientists with disabilities have to work harder than scientists without disabilities do (Serrato Marks and Bayer 2019). For example, scientists are expected to move across countries during their career or to attend international conferences, which can be difficult and very expensive for certain disabilities (Schaal 2018). Moreover, researchers with disabilities are compared directly in terms of their research outputs with non-disabled scientists while trying to secure a new post or applying for research grants (Kosanic et al. 2018). This puts even more strain on the well-being of scientists with disabilities as they are placed in a treadmill-wheel, a non-winnable and non-equal academic race. Furthermore, during pandemics (i.e. COVID-19), work of scientists with disabilities might “experience disproportionate disruption to their work” showing the necessity for granting bodies to allow extensions (Niedernhuber et al. 2021). Disruptions not solely related to the Global pandemic can affect scientists with disabilities over time, affecting academic career progressions. A “rigid picture of ‘ideal academic’” (Brock 2021) is still in place and caring actions to switch “from metrics to merits” are crucial to stop re-enforcing unhealthy and unequal competition (Care et al. 2021).

Also importantly, a feminist ethos of care practice reminds us that diversity is highly beneficial for academia (Schaal 2018) because it allows institutional progression and exclusion of traditional leadership models (Care et al. 2021). As disability tends to be ignored when organizing conferences, workshops and meetings (Blackman et al. 2020; Serrato Marks 2018), a caring sustainability science needs to consider accessibility as an essential component of the diversity strategy when organizing meetings (Goring et al. 2018; Serrato Marks et al. 2021). In doing so, organizers of conferences as well as scientific societies should engage scientists with disabilities to organize panels, forums or discussion groups around ableism, disabilities and research (Serrato Marks et al. 2021). Future conferences of Transdisciplinary Sustainability Science, such as the International Transdisciplinarity Conference or the conference of the Programme of Ecosystem Change and Society (PECS), can benefit from such conversations which might contribute to foster justice and equity and provide insights on how to engage with non-academic research partners who have different disabilities.

People with disabilities are often not considered as a stakeholder group to engage with when conducting Transdisciplinary Sustainability Science. To overlook people with disabilities when addressing sustainability problems can entail a major setback since they are more likely to be exposed to extreme climate events, loss of natural resources and infectious diseases (Kosanic et al. 2019). In addition, people with disabilities are more likely to experience limited access to resources and services to respond effectively to environmental and climate change (Kosanic et al. 2019; Larrington-Spencer et al. 2021). Therefore, a feminist ethos of care practice should include disabled populations as relevant stakeholders and research partners in any sustainability research project that deals with environmental change and environmental justice. In doing so, transdisciplinary methods should be adapted to accommodate different disabilities. If knowledge, needs and interests of people with disabilities are not included during the research, it is doubtful that the sustainability solutions can support the future of this community.

A thoughtful and caring culture of inclusion and understanding of the different disabilities is needed. Transdisciplinary Sustainability Science cannot afford the lack of such inclusive culture because sustainability and just solutions must include minorities, including disabled populations. It is time to respect, recognize and consider scientists with disability and non-academic research partners in Transdisciplinary Sustainability Science.

*Thinking-with* is therefore committed to collective action and the belief of “the everyday as political practice” (Bayfield 2019: 2). Collective research practices grounded in a feminist ethos of care are capable of radically restructuring our academic institutions as nurturing working and learning environments. For example, in October 2019, the Care Operative started as a leadership collective experiment that aims to provide “a reflexive, inclusive and caring space for members as (they) pursue our mission to collectively explore, embody and lead transformational sustainability research and practice” (Care et al. 2021). During the COVID-19 pandemic, the Care Operative has used virtual meetings to develop a collegial support system that allows the collective to provide peer support to deal with the challenges of care duties or conducting research in foreign contexts (Care et al. 2021; The Care Operative 2020). Inspired by the notion of a feminist-infused ‘slow scholarship’ (Berg and Seeber 2017; Mountz et al. 2015), we suggest an inclusive, but not exhaustive, list of further collective practices that may help to cultivate caring environments (Box 3).

Box 3 List of practices that may help to cultivate caring environments in academia and beyond

- Take time to meet with colleagues to discuss and develop ideas (Mountz et al. 2015).
- Talk about your private life and how intertwined it is with professional life (Mountz et al. 2015).
- Share expertise on academic literaturs, methodologies and writing and give each other advice in terms of career advancement (Bayfield et al. 2019).
- Collectively commit to email-free weekends and evenings to take care of worlds outside of academia (Mountz et al. 2015).
- Practice collective writing in nourishing environments, e.g. writing retreats (Bayfield et al. 2019). Here, we should note the potential setback to foster caring practices when these writing retreats happen over weekends. These practices might result in ever-greater intrusions of academia into personal lives, where ‘our work is our life’.
- Motivate each other to proceed after setbacks (Bayfield et al. 2019).
- Realise your ability of humour to ease seemingly unresolvable situations (Thiem and Katz 2015).
- Appreciate and celebrate each other’s successes, irrespective of how small or big they may seem (Macy and Johnstone 2012).
- ‘Grieve-with’ others about the destruction of our loved planet and aggravating social inequalities (Haraway 2016).
- “[R]esist intensified pressures to do it all” (Mountz et al. 2015: 1248), e.g. as members of underrepresented groups in academia (women, people of colour, people with disabilities or indigenous people) you can say ‘no’ to committee work.
- Develop a peer mentoring scheme in which problems can be expressed without fear of being penalised by someone in a position to support your career advancement (Bayfield et al. 2019).
- Imagine success differently, celebrate slowness as an essential component of good scholarship and develop ambitions beyond academia (Bayfield et al. 2019; Paasche and Österblom 2019).
- Overcome narrow quantitative evaluations of academic work (for example, counting for collaborations forged with academic and non-academic partners) and make a wider range of work count in decisions about hiring, raises, graduate student advancement and tenure and promotion (Mountz et al. 2015).
- As tenure, grant, or manuscript reviewers, reward ‘care-full’ research projects which foster sensitive, inclusive, and respectful modes of working and provide resources for cultivation of inner health and well-being (Care et al. 2021; Grummell et al. 2009; Ives et al. 2020).

## Taking care of non-academic research partners

Seen through the lens of a feminist ethos of care, transdisciplinary knowledge production is essentially about creating and nurturing relationships, not only within a researcher collective, but also with non-scientific actors (Fam et al. 2017). By “explicitly valorising the collective webs one thinks with” (Puig de la Bellacasa 2012: 202), these relationships should be based on the principles of participation, inclusion and acceptance of the validity of diverse perspectives, so that context-specific knowledge and pathways towards critical-emancipatory futures can be produced (Norström et al. 2020). Following the feminist notion of situated knowledges, it becomes crucially important that we ensure that participants with a range of skills and multiple types of knowledge and expertise take part in research projects to tackle a multidimensional sustainability problem holistically (Lang et al. 2012). Apart from resourceful (non-)academic actors, it is particularly important that we ensure the participation of disadvantaged people (as will be discussed more thoroughly in section *thinking-for*). For example, this might become possible through financial compensation for their work (e.g. travel expenses, food for participants with special dietary needs, childcare costs, or compensation for loss of earnings) or, in the case of people with disabilities, by providing assistance on site and barrier-free access (Bergold and Thomas 2012).

The overarching goal of a feminist ethos of care is to contribute to capacity building and empowerment on part of our non-academic partners. We need to act as engaged partners who sincerely show that we care for them and therefore aim towards facilitating strong forms of collaboration (Hesse-Biber and Piatelli 2012). By developing and nurturing diverse communication and interpersonal skills, we are required to stimulate participation, promote dialogue and foster relationships to build a collaborative research team (Fam et al. 2017). As facilitators of meaningful collaboration across different social groups, we should refrain from scientific communication habits and translate relevant theories and concepts by means of visual products and media or everyday terms (Lang et al. 2012; Norström et al. 2020). To make our communication skills more effective, we can attend specific seminars and workshops. For example, the National Institute for Science Communication (NaWik) based in Karlsruhe, Germany, offers trainings to improve scientists’ communication skills and develop meaningful visual products for non-academic partners (NaWik 2021).

Importantly, we are required to engage non-academic partners as co-researchers within an organisational project structure that is guided by the principle of joint leadership, encompassing shared rights and obligations (Lang et al. 2012). For example, empirical place-based studies on the plural valuation of nature show that those research processes that are collaborative, articulate different knowledge systems, and trigger shifts in power have led to sustainability and justice outcomes (Zafra-Calvo et al. 2020). By contrast, those research processes that do not engage with and contest power relations and do not reflect on the research process itself with non-academic partners have not achieved sustainability outcomes (Osinski 2021a). Aiming for empowering cooperation demands that we give encompassing decision-making authority to partners, which will play out differently in each research setting (Ghosh 2020; Moriggi et al. 2020a, b). It is important to transparently discuss and agree on how co-determination and shared responsibility are to be realised—not least to counter stereotypes of non-academic partners who expect that we tell them how the project should be run (Di Giulio et al. 2016). We need to assess regularly whether partners feel they have sufficient opportunities to participate (Norström et al. 2020) and make sure to present co-determination as a topic that we discuss repeatedly and reflect on in the project (Fritz and Meinherz 2020).

Concluding, *thinking-with* aims at fostering mutual trust, dialogic relationships and understanding, long-term commitment, participatory settings, empowerment and capacity building within pluralistic research collaborations. Within these collaborations, it is crucial to ensure “accountable knowledge construction that does not negate dissent” (Puig de la Bellacasa 2017: 79).

## Dissenting-within

### Engaging with conflict and difference

Diverse positionalities (see Box 1) of research participants can overall provide a more holistic account of the research problem but render conflicting viewpoints and interests inherent to transdisciplinary projects (Dietz et al. 2021; Siebenhüner 2018). Appreciating conflict arising from the diversity, difference, and contradictions of both academic and non-academic positionalities is thus highly important (Puig de la Bellacasa 2017: 78). This requires us to give up notions of win–win solutions following a harmonious, smooth process and accept that trade-offs are unavoidable in transdisciplinary projects (Dietz et al. 2021; Hofmeister 2017). As Gottschlich and Katz (2019: 13–14) state, “[i]t is not about comfort, self-affirmation, home and the stabilization of identity, but about hard work that involves being questioned by others and yet caring for these others”. We as caring researchers with a commitment to reciprocity should then undertake the challenging task of establishing, facilitating, and participating in controversial discussions with praxis partners.

Jointly defining rules of engagement at the beginning of the project can facilitate the creation of a productive culture of a caring dispute (Atkinson-Graham et al. 2015). Also importantly, utilising bridging concepts or boundary objects might be helpful to reveal differences, which may then be transformed into a pluralistic understanding of the problems whilst also widening the spectrum of available solutions (Hofmeister 2017). On a regular basis, if the project design allows for it, we can host interim workshops (Dietz et al. 2021), in which we openly discuss divergent and opposing needs, interests and expectations and might then decide to adapt the research agenda (Di Giulio et al. 2016; Norström et al. 2020). For example, in the study by Galafassi et al. (2018) observers took notes during workshops about whether and how they experienced conflicts. After the workshops, these notes were then shared with the leading research team to provide multiple perspectives on conflictive situations. The development and integration of self-managed conflict management programmes (Löhr et al. 2016) and external support from consultants/mediators who might help us disclose and moderate conflicting objectives and interests might help to create a culture of caring dispute (Dietz et al. 2021).

Overall, in these conflictive discussions, we are required to be open to negotiation and listen to critique from our partners (König 2017). “[T]o know and care about the way they think” (Puig de la Bellacasa 2017: 82) also involves considering that positional differences between individuals who belong to different social groups lead to interactions, which are inevitably unequal (Young 1997; Clifford 2013). This means that we cannot immediately and entirely understand an unfamiliar social world and that we must, therefore, wait to learn and gain a gradual understanding by carefully listening and engaging with the other person (Edwards and Mauthner 2012). Consequently, to authentically engage with the perspective of others who we might not understand in the first place given their different social positioning, we should promote the important feminist practice of reflexivity.

### Interrogating positionalities and power relations through reflexivity

The inherently conflictive nature of transdisciplinary research projects results from the diversity of social positions of our partners and us researchers. *Dissenting-within*, hence, requires that we recognise our ‘withinness’, that is, “[our] situatedness in the production of knowledge” (Puig de la Bellacasa 2017: 80) and demands that we pursue the practice of *reflexivity*. Reflexivity requires us to radically reflect—throughout the whole research process—on both the power relations that suffuse the project and, more personally, our own positionality, our classed, racialized, gendered and political backgrounds, theoretical positions, normative assumptions, beliefs and world views (Hesse-Biber and Piatelli 2012; Lumsden 2019; Norström et al. 2020). Research collaborations are always influenced by power dynamics among researchers and between researchers and non-academic actors, which are multi-faceted and changing depending on context and research stage (Hesse-Biber and Piatelli 2012; Turnhout et al. 2020). For example, Fritz and Binder (2020) outline in their qualitative meta-analysis of five transdisciplinary sustainability research projects in Germany how funding agencies, researchers, and non-academic actors exert different forms of power (instrumental, structural, and discursive) over actor selection, agenda setting, and rule setting. These different forms of power work in different ways and can be both productive, i.e. facilitate empowerment (power to) and processes of collective learning (power with), and repressive, i.e. dominate actors, structures, and discourses (power over) (Fritz and Meinherz 2020).

Based on reflexivity, *dissenting-within* accordingly calls on us to make the hierarchical power relations that underpin conflicts of interest a subject of discussion with our research partners and examine more thoroughly, both how and on whose terms, knowledge is integrated (Pettibone et al. 2018). This includes collectively becoming aware of and explicitly acknowledging differences and hierarchies in the status and effectiveness of different forms of knowledge, as well as differences in status among the knowledge producers (Hofmeister 2017; Turnhout et al. 2020). To do so, Fritz and Meinherz (2020) propose a seminal list of empirical questions that can help us trace interwoven productive and repressive power dynamics in different research phases. Kohl and McCutcheon (2015) suggest to apply the ‘kitchen table reflexivity’, this is, how everyday talk and informal encounters at different research stages among researchers, but also between researchers and non-academic actors, can facilitate a thorough understanding of our shifting positionalities and build long-term trustful relationships.

By calling on us to make our normativity (see Box 1) and value judgements explicit, reflexivity as part of the mode *dissenting-within* also reminds us that we can only take care of a *particular* kind of sustainability and a *particular* community to be able to effectively and responsibly deal with their problems (Puig de la Bellacasa 2017: 80). This mode of thinking is a call to engage productively with conflict and to commit openly to specific normative struggles (Haraway 2016). *Thinking-for* encompasses this commitment and appeals to us to produce knowledge that benefits members of marginalised groups, mitigates power imbalances, and challenges systems of domination.

## Thinking-for

### Building upon marginalised knowledges via feminist standpoints

*Thinking-for* emphasises that caring knowledge production is intrinsically connected with an “awareness of oppression and with commitment to neglected experiences that create oppositional standpoints” (Puig de la Bellacasa 2017: 61). Following Puig de la Bellacasa, caring knowledge production must therefore draw on feminist standpoint theory—a debated feminist epistemology (i.e. ways of knowing) and methodology (i.e. way of doing research) that emerged from second-wave feminist thinking and points to the interrelatedness of power and knowledge production. It draws on the notion of ‘situated knowledges’, highlighting that our social position shapes and limits our knowledge through our experiences (Intemann 2016). Given that experiences are socially situated, feminist standpoint theorists claim that knowledge is cultivated from a particular standpoint.

A *standpoint* can be understood as a collectively achieved “oppositional consciousness […] based on solidarity and commonality defined against the interests of dominant classes and groups” (Snyder 1995: 95). A precondition to achieve a feminist standpoint is the political commitment to analyse, critique and transform systems of oppression that influence transdisciplinary inquiry. If we want to achieve a feminist standpoint, we must be committed to produce “knowledge for marginalised groups to counteract, remove, or minimize the ways in which oppressive systems limit the health, well-being, or life prospects of the members of these groups (including their ability to participate in the production of knowledge)” (Intemann 2016: 268).

We are required to work with deprived communities to set research agendas and define research problems, because, first, they are disproportionately affected by sustainability problems while bearing little responsibility for the existence of these problems (Kaijser and Kronsell 2014; Nixon 2013; Scheidel et al. 2020). For example, the Environmental Justice Atlas (2021) has documented the numerous struggles of marginalised communities across the world to defend their livelihoods from extractive activities and damaging projects, such as mining, fracking, dams, nuclear waste, and tree plantations. This research project makes visible the heavy burden that is placed on the shoulders of deprived communities in the forms of expulsion, expropriation, criminalisation, and physical violence. In light of the painful lack of (inter)national legal regulations that hold the perpetrators of socio-environmental violence accountable for their crimes, the project also acknowledges the communities’ rights to use and fight for their land (Scheidel et al. 2020; Tran et al. 2020).

Second, members of deprived communities might have unique insights that reflect their lived experience as ‘insiders-outsiders’ (Hill Collins 1991; Turnhout et al. 2012). That is, not only do marginalised people have to understand the assumptions of epistemically privileged groups to successfully navigate the world, but their lived realities simultaneously create a dissonance between these dominant views and their own alternative understanding about how the world works. This insider–outsider perspective might productively reveal problems to be explained, limitations of current assumptions, models, theories, methodologies and research questions and identify alternatives that may offer valuable resources for reinterpreting what is known and generating new knowledge (Intemann 2016; Osinski 2021b). To “mov[e] subjugated knowledge from the margin to the centre of social inquiry” (Hesse-Biber and Piatelli 2012: 568) is not only essential to find new solutions to sustainability challenges (Nagendra et al. 2018), but also intricate and controversial.

There are pitfalls that attempts at thinking from subjugated positions might entail. For instance, we may confuse ourselves with the spokespersons “using marginalized ‘others’ as arguments, or falling into a fascination with the inspiring experiences of ‘the marginal’ or the oppressed” (Puig de la Bellacasa 2017: 85). These actions are an expression of epistemic violence (Spivak 1988) which occurs if we, for instance, analyse and work on the alleged or actual problems of people whom we consider as belonging to a marginalised group. In such circumstances, we might fail to take into account the categorisations and interpretations of those being affected and instead impose our own understandings. *Speaking for* marginalised ‘others’ silences them, denies them the status of epistemic subjects and instead, degrades them, reducing them to objects who are merely known by others. Epistemic marginalisation thus accompanies and legitimises their material subordination (Bowell 2011).

### Countering epistemic violence within and beyond academia

Following Spivak (1988), a postcolonial feminist thinker, we are called upon to pursue a cautious, reflexive approach to the problem of how to carefully work with and represent non-academic research partners. This requires creating spaces in which we *speak to* and *with*, not *for* marginalised partners. Instead of appropriating (see Box 1) marginalised voices to pursue our own academic advancement, we should actively support these communities in achieving their own goals and patiently try to learn from them to re-construct their specific perspectives and needs. An important step to achieve this goal is to ensure the actual presence of marginalised people in all stages of the research, and not just introduce simple verbal or written testimonies, or video presentations into the process when it is convenient (Osinski 2021b). As members of deprived communities most often experience multiple barriers to attend participatory processes outside their living environment (e.g. Adams et al. 2020; Montesanti et al. 2016; Ward et al. 2018), face-to-face interaction through regular visits of or even temporary stays at these communities to share their everyday life is crucial (Kohl and McCutcheon 2015). Financial compensation in various forms, as already mentioned in the section thinking-with, is another effective measure to ensure inclusive participation (Bergold and Thomas 2012).

Given that marginalised people often share “deeply engrained histories of mistrust resulting from neglect or social exclusion” (Dedeurwaerdere 2014: 95), we must create spaces of trust and security to *speak to* and *with* them (Osinski 2021a). We should set up a contract at the beginning of the project which involves that everything that they say or write is handled with confidentiality, respect, and reflexivity (Osinski 2021b). Moreover, when other powerful or dominating stakeholders are present, marginalised participants might, understandably, not be willing to contribute their ideas because they fear multiple negative consequences. In this case, methods like focus group discussions, which are often used in Environmental Justice research to enable diverse opinions and to shed light on the subtle ways in which unequal power relations are structured in group settings, might need to be complemented with individual private interviews. They can minimise the potential for groupthink, false consensus, or hesitation at sharing one’s critical or alternative perspective (Graham et al. 2017).

Also importantly, trust-building to empower disadvantaged community members necessitates innovative communication methods (Godinot and Walker 2020). For example, Masterson et al. (2018) used photovoice to get a more holistic picture of the multi-faceted dimensions of human well-being in changing socio-environmental systems. Photovoice not only helped to overcome social and cultural barriers of communication but also facilitated the participation of marginalised women. Following the Merging Knowledge Approach, developed by the international movement ATD Fourth World (ATD Fourth World 2021) to combat poverty through the active engagement of people experiencing poverty, the transdisciplinary project “The Hidden Dimensions of Poverty” (Bray et al. 2019) included a pedagogical team and discussion facilitators. They supported disadvantaged participants in expressing themselves in their own words, reflecting on their experience, and understanding the other participants, and also helped researchers and other professionals to make their formulations more comprehensible and to reflect on their biases (Osinski 2021b).

For critically interrogating our epistemic biases—as part of the practice of reflexivity—we should understand and reveal how systems of domination, such as classism, sexism, racism, and ableism, shape and limit our research questions, methodological decisions, conceptual frameworks, models, assumptions, or interpretations of data (Harding 2004; Intemann 2016). For instance, in their analysis of the World Bank’s Consultation with the Poor, Cornwall and Fujita (2012) highlighted that there were dominant conceptual categories in the methodology, such as ‘vulnerability’, ‘the poor’, and ‘social exclusion’, that limited the space for participants to explore their own categories and meanings. This study shows that instead of portraying marginalised people as individuals in a continuous state of despair and suffering (Osinski 2021b), we should recognise their resourcefulness, multiple skills and strengths, and creativity (Bray et al. 2020).

Moreover, to consider the heterogeneity of a marginalised community and examine the ways a problem may manifest differently for individuals within it, we should draw on intersectional (see Box 1) Environmental Justice approaches (Malin and Ryder 2018; Menton et al. 2020). For instance, Vickery (2018) examined how discrimination against people who were homeless during the Colorado floods in 2013 manifests differently for individuals within this unprivileged group, based on other identity markers, such as their gender, and physical or mental capacities. Intersectional approaches ensure that the framing of research problems, choice of methodologies, selection of data and interventions developed are more likely to be responsive to and effective for the needs of marginalised, but internally diverse groups (Osborne 2015; Malin and Ryder 2018). In this regard, conflicting values and interests among differently positioned members within marginalised groups, particularly in terms of power, should be discussed (Intemann 2016).

Critically negotiating questions of power is also needed within academia. Sustainability scientists who are committed to *thinking-for* should question how their privileged position can help to make the arena of academia more accessible for people who face continuous gendered (Winslow and Davis 2016), racialised (Laland 2020), classed (Lee 2017), heteronormative (Mintz and Rothblum 2013) and ableist (Dolmage 2017) structures of oppression. For instance, women of colour, working-class women, women who identify as non-cisgender and queer women are particularly underrepresented in academia and face severe challenges in pursuing academic careers (Bayfield et al. 2019). *Thinking-for* encourages us to challenge elitist exclusions through radical involvement in personnel policy decisions, thereby countering social discrimination (Mountz et al. 2015; Thiem and Katz 2015). Given their insider–outsider position, these marginalised academics might be better able to identify the underlying assumptions and norms that drive and shape the power dynamics within academic institutions and their research practices. This might enable them to pose challenging questions about the social-political structures that perpetuate and produce epistemic violence (Bowell 2011) (see also Box 2).

To conclude, *thinking-for* requires us to embrace the ethico-political commitment to critically expose the ways in which power structures limit and shape knowledge production and to offer a counter-approach that challenges such systems of oppression.

## Outlook for practicing caring knowledge production in transdisciplinary sustainability science

We highlight that the examined three modes of caring knowledge production can be regarded as principles that formulate a quality standard for Transdisciplinary Sustainability Science. There is no ideal path for the integration of these three modes into structures, processes and actions in a transdisciplinary research project, given the diverse challenges at hand, the actors involved, the social-political contexts and the scale of the project. We as researchers should aim to generate context-specific caring action, without necessarily making one way of caring the template for other kinds of caring. This also implies that not all suggestions we proposed here can be implemented at the same time and that there will be trade-offs between different actions depending on the context.

To minimise the trade-offs that are inherent to processes of caring knowledge production, structural changes in academic institutions that combat the ever-growing marginalisation of care through ‘fast science’ (Stengers 2017) are necessary. Fast science is intrinsically interwoven with “competitive evaluation, publication in high-impact-factor journals, inbreeding review by peers and industrial capture of financial research resources” (Perezgonzalez et al. 2018: 1). If a feminist ethos of care is to be established in research institutions, there is a need for funding policies and incentive structures that encourage pluralistic, open-ended, and power-critical projects to foster long-term emancipatory sustainability change (Gottschlich and Katz 2016; Katz et al. 2015). This also includes a fundamental restructuring of time economies within academia (Reisch 2015). Only if we have the financial and time resources, are we able to build caring relationships with our colleagues, our research partners outside academia and ourselves (Hofmeister 2017; Ghosh 2020). In doing so, we can far better facilitate reciprocity, processes of mutual learning and experimental projects, as well as engage in practices of reflexivity to reveal hidden power mechanisms and the perpetuation of oppressive relations (Gottschlich and Katz 2016).

In this regard, we must apply this critical lens to ourselves as transdisciplinary sustainability scientists, and in so doing acknowledge that our examinations arise from a position of relative privilege. As three white women, academically socialised at an almost white university with Western scholars, we are aware of our complicity with hegemonic regimes and the silences that we produced over the course of this work. For instance, in this paper we could have included more postcolonial or decolonial voices, which can provide highly productive contributions to a power-critical Transdisciplinary Sustainability Science, particularly in transnational research collaborations (Chilisa 2017; Ghosh 2020; Schmidt and Neuburger 2017). What we proposed as a feminist ethos of care may reflect a predominantly privileged perspective on care, which might have prevented us from seeing and including other important perspectives. Moreover, it is important to emphasise that the notion of the interconnectedness of all beings did not originate from feminist thought. It has a long tradition in diverse non-Western philosophies, such as indigenous ontologies, Buddhism and Confucianism, which can all contribute to a further development of a transformative Sustainability Science. Our perspective is partial, and in this paper we could only consider some thoughts and ideas and not others (Haraway 1988). We see this, however, not as a hindrance, but as an asset of our work because we want to challenge distorted views on academic objectivity and, most importantly, invite readers to discuss, apply, and develop the suggested framework further.

## Conclusion: relations of thinking and knowing require care

We argued that incorporating a feminist ethos of care can contribute to the extension of Transdisciplinary Sustainability Science’s transformative potential. Caring knowledge production is grounded in the fundamental belief that challenging power structures is the only viable answer to this un-sustainable world. It involves the active acceptance of responsibility for, and commitment to, building research relationships in which trust, strong forms of collaboration, transformative dispute and critique of oppression, are core principles. *Thinking-with* demands that we work with relational concepts and ontologies, affectivity and diverse creatures and people. It also encourages us to foster mutual trust, dialogic relationships and understanding, long-term commitment, participatory settings, empowerment, and capacity building within pluralistic research collaborations. *Dissenting-within* does not only require that we endure and embrace differences and conflict, but also that we practice reflexivity to become aware of, and to challenge, underlying paradigms, and hierarchies. *Thinking-for* requests us to think with and for marginalised others in a directional, rather than a representational sense, whilst critically interrogating our own privileges and complicity within hegemonic knowledge regimes.

Drawing on the feminist concept of care, we propose that Transdisciplinary Sustainability Science might be able to gain radical perspectives and embark down innovative avenues that can enact change for more sustainable futures. Producing knowledge for these futures requires not only a new scientific self-identity, but also fundamental changes in the organisational and institutional framework of research. If Transdisciplinary Sustainability Science wants to extend its transformative potential and contribute to the achievement of sustainable development, there is no way around deep structural transformation that radically challenges seemingly unshakeable structures of dominance and power in academia, society and governance.
